# Open-Cell AlSn6Cu-SiC Composites: Fabrication, Dry-Sliding Wear Behavior, and Machine Learning Methods for Wear Prediction

**DOI:** 10.3390/ma16186208

**Published:** 2023-09-14

**Authors:** Mihail Kolev, Ludmil Drenchev, Veselin Petkov, Rositza Dimitrova, Daniela Kovacheva

**Affiliations:** 1 Institute of Metal Science, Equipment and Technologies with Center for Hydro- and Aerodynamics “Acad. A. Balevski”, Bulgarian Academy of Sciences, Boulevard Shipchenski Prohod 67, 1574 Sofia, Bulgaria; ljudmil.d@ims.bas.bg (L.D.); veselin.petkov@ims.bas.bg (V.P.); rossy@ims.bas.bg (R.D.); 2 Institute of General and Inorganic Chemistry, Bulgarian Academy of Sciences, 1113 Sofia, Bulgaria; didka@svr.igic.bas.bg

**Keywords:** AlSn6Cu-SiC, coefficient of friction, liquid-state processing, wear prediction, machine learning

## Abstract

Open-cell AMMCs are high-strength and lightweight materials with applications in different types of industries. However, one of the main goals in using these materials is to enhance their tribological behavior, which improves their durability and performance under frictional conditions. This study presents an approach for fabricating and predicting the wear behavior of open-cell AlSn6Cu-SiC composites, which are a type of porous AMMCs with improved tribological properties. The composites were fabricated using liquid-state processing, and their tribological properties are investigated by the pin-on-disk method under different loads (50 N and 100 N) and with dry-sliding friction. The microstructure and phase composition of the composites were investigated by scanning electron microscopy, energy-dispersive X-ray spectroscopy, and X-ray diffraction. The mass wear and coefficient of friction (COF) of the materials were measured as quantitative indicators of their tribological behavior. The results showed that the open-cell AlSn6Cu-SiC composite had an enhanced tribological behavior compared to the open-cell AlSn6Cu material in terms of mass wear (38% decrease at 50 N and 31% decrease at 100 N) while maintaining the COF at the same level. The COF of the composites was predicted by six different machine learning methods based on the experimental data. The performance of these models was evaluated by various metrics (R2, MSE, RMSE, and MAE) on the validation and test sets. Based on the results, the open-cell AlSn6Cu-SiC composite outperformed the open-cell AlSn6Cu material in terms of mass loss under different loads with similar COF values. The ML models that were used can predict the COF accurately and reliably based on features, but they are affected by data quality and quantity, overfitting or underfitting, and load change.

## 1. Introduction

The wear behavior of Al-based metal matrix composites (AMMCs) is essential for the performance and longevity of these materials, especially in applications where they are subjected to high loads, speeds, and temperatures, such as in the aerospace, automotive, and biomedical industries [1,2,3]. AMMCs are a type of composites that have a metal matrix reinforced with continuous/discontinuous fibers, particulates, and whiskers [4]. AMMCs have some advantages over conventional metals, such as higher strength, stiffness, wear resistance, and thermal conductivity [5,6]. However, AMMCs also face some challenges in terms of their sliding contact performance. The presence of reinforcement particles can have different effects on the wear mechanisms of composites, depending on the type, size, shape, distribution, and orientation of the particles [7]. Moreover, the reinforcing particles can cause abrasive or adhesive wear to the composite and the counterface [8,9]. Therefore, AMMCs need to be carefully designed and optimized to achieve a balance between their mechanical and wear properties [1]. It is important to understand how AMMCs perform under sliding contact, which is one of the main sources of wear in these applications. Several studies have investigated the effects of different types of reinforcement particles on the tribological properties of AMMCs. For instance, B_4_C particles were used as reinforcements in AA6061 by [10] and Gr was used by [11], and the authors found that the reinforcement improved the hardness and wear resistance of the AA6061-B_4_C composite and the AA6061-Gr composite, respectively. Al_2_O_3_ particles were used as a reinforcement in AA7075 by [12], and the authors reported an improved volume loss and compared to the base alloy. SiC particles were used to reinforce AA6061 by [13], and the results indicated an improved wear rate of the composite compared with the base alloy. The wear behavior is influenced by different factors such as the microstructure, composition, porosity, pore size, reinforcement type and content, sliding speed, temperature, load, and environment [14,15]. Therefore, it is important to study how these factors affect the wear behavior of AMMCs and to find reliable methods for estimating and optimizing their wear behavior.

The manuscript in [13] describes the fabrication and characterization of SiC/Al 6061 composites with high SiC contents using pressure-assisted infiltration. It also investigates the wear behavior of the composites under different SiC contents and shows that a higher SiC content (75 wt.%) leads to a lower wear rate and coefficient of friction (COF). Hassan Sharifi et al. [7] fabricated Al-Mg/SiC-Al_2_O_3_ and Al-Mg/Al_2_O_3_ by pressureless infiltration and conducted dry sliding pin-on-disk method tests. The results indicated that the test specimens with higher densities and smaller cell sizes had lower wear rates. The authors in the study in [16] fabricated Al-Cu composites with different contents of TiC-SiC (2–8 wt.%), which served as a reinforcement, by employing stir casting and studied their wear properties. The wear behavior of the AMMC indicated that the optimal wear resistance of the material was obtained by the addition of 10 wt.% of reinforcement. The authors in [17] used a combination of the stir-casting and squeeze-casting methods to produce Al-based composites with SiCp (20–30 wt.%) and to study their microstructural, mechanical, and wear properties. The results showed that the dual casting processes improve the SiCp distribution, reduced the porosity, and enhanced the mechanical and wear performance compared to using only the stir-casting technique. The composites with 30 wt.% of SiCp had the highest hardness, lowest wear rate, and lowest COF. Shaikh et al. [18] used powder processing to fabricate Al-Si composites with SiC (1, 3, and 5 wt.%), and after obtaining the results of dry-friction tests, they conclude that the composites with 5 wt.% showed the lowest COF and a significantly decreased mass wear compared to the rest of the composites.

Predicting the wear behavior of open-cell AMMCs accurately is important because it can help to improve their performance, reliability, and durability in industrial applications. Inaccurate predictions can lead to costly failures and reduced efficiency in these applications. For instance, excessive wear can cause dimensional changes, surface damage, material loss, and increased friction in these composites. This may lead to higher energy consumption, lower functionality, more maintenance costs, and a reduced service life for these composites [19,20]. Therefore, providing some context on the importance of the tribological behavior and wear prediction of open-cell AMMCs can help to motivate further research on these composites and their potential applications.

The study in [19] relates the use of machine learning (ML) algorithms to optimizing the wear behavior of ball bearings by reducing the severe vibrations caused by defects. Fatih Aydin et al. [21] fabricated ZK60/CeO_2_ with different contents of reinforcement materials (0–1 wt.%) and tested their wear behaviors under different loads (5–30 N). The study also used five ML algorithms to predict the wear behavior based on a limited dataset and compared their performance using statistical measures. The study relates the use of ML algorithms to optimizing the wear behavior of the composites by identifying the best model and the optimal parameters for reducing the wear rate and COF. The authors of [22] used a neural network model modified with a particle swarm optimizer to predict the tribological properties of an Al-TiO_2_ nanocomposite based on its experimental data and features. The model showed a high accuracy for different wear loads and composite compositions for the coefficient of friction and wear rate. The authors of the article [12] obtained an Al-based composite reinforced with Al_2_O_3_ by hot pressing and studied the effect of the ceramic size in relation to the wear behavior. Using four machine learning methods to predict and compare the volume loss of the composites based on the experimental data, the study found the best model (extreme learning machine) and the optimal parameters (particle size, load, and speed) for optimizing the wear behavior of the composites by minimizing the volume loss and improving the wear resistance. Hasan et al. [20] employed ML algorithms to understand and predict the wear and COF of Al-based alloys based on their processing procedure, material properties, heat treatment, and wear test variables. The study applied five different ML algorithms to experimental tribological data and compared their performance and feature importance. The study relates the use of ML algorithms to optimizing the wear behavior of Al alloys by finding the best models (random forest for wear rate and k-nearest neighbors for COF) and the most influential parameters (hardness, normal load, and sliding speed for wear rate; sliding distance and hardness for COF) for minimizing the wear and friction of the Al alloys. The COF of open-cell composites with AlSi10Mg serving as a matrix with different reinforcements was predicted in previous studies. The random forest (RF) model [23] and the support vector regression (SVR) model [24] were used for composites reinforced with SiC, and the extreme gradient boosting (XGBoost) model [25] was used for composites reinforced with Al_2_O_3_.

This study aims to fabricate open-cell AlSn6Cu-SiC composites using the liquid-state processing and replication methods. This study also aims to investigate the wear behavior of these composites under different loads using the pin-on-disk method and to measure their mass wear and COF as quantitative indicators of their tribological behavior; to characterize the microstructure and phase composition of the composites using scanning electron microscopy (SEM) images, energy-dispersive X-ray spectroscopy (EDS) analysis, and X-ray diffraction (XRD) patterns; to predict the COF of the composites using six different ML methods; and to analyze the feature importance for predicting the mass wear and COF of the composites and unreinforced materials using random forest (RF) regressors.

This research expands upon previous studies on open-cell AMMCs with different reinforcements and pore sizes, which were fabricated and tested using similar methods. For example, open-cell AlSi10Mg-Al_2_O_3_ (with pore sizes of 800–1000 μm and 1000–1200 μm) were produced by employing the replication method and using liquid-state processing, and their wear behavior was tested by the pin-on-disk method at a sliding velocity of 1.0 m∙s^−1^ and an applied load of 50 N [26]. Similarly, open-cell AlSi10Mg-SiC (with pore sizes of 800–1000 μm) was tested at a load of 50N, and its COF was predicted by three ML methods [27]. Moreover, infiltrated open-cell AMMCs with a tin-based Babbitt alloy were also investigated for their tribological properties [28,29]. Therefore, this study aims to explore the effects of using a different alloy (AlSn6Cu) as a matrix, incorporating SiC particles as a reinforcement, and applying six ML methods for predicting the COF of open-cell AlSn6Cu-SiC composites as well as analysing the feature importance for predicting the mass wear and COF of the composites and unreinforced materials.

## 2. Materials and Methods

### 2.1. Production Method and Materials

The open-cell AlSn6Cu-SiC composites were fabricated by squeeze casting. The materials used for the fabrication of the open-cell AlSn6Cu-SiC composites were an aluminum alloy (AlSn6Cu), which was used as the matrix (see its composition in Table 1), silicon carbide particles ranging from 300 to 400 μm serving as the reinforcement, and NaCl particles ranging from 1000 to 1200 μm, which were used for the salt preform preparation by the replication method [30]. AlSn6Cu is an alloy intended for the production of solid bearings. Al compositions for bearing applications have Sn as the main element that is added to them, along with other elements such as Cu, Ni, and Si, to improve their mechanical and tribological properties. The soft Sn phase can help to create a protective layer on the contact surface, which extends the bearing life [31,32,33]. This was the reason for choosing AlSn6Cu as the matrix of the composite, as it can provide good friction and wear performance under dry-sliding conditions. The SiC particles were chosen for their high hardness, strength, and wear resistance. The content of the reinforcement (5 wt.%) was based on preliminary studies related to dry-friction wear behavior [18,27].

The fabrication process involved three steps. First, NaCl preforms were prepared by using the replication method. A 3D powder blender was used to homogenize a mixture of NaCl particles as soluble space holder, 5 wt.% SiC particles, and 6 wt.% water. The mixture was then pressed into a steel cylinder with a 1.5 MPa pressure to obtain the preliminary preform. The obtained green compacts were dried in a 200 °C furnace for 2 h to eliminate moisture. The salt-leachable preform was obtained by sintering the green dried compacts at 800 °C ± 1 °C for 1 h and then cooling them at room temperature. Second, the NaCl preforms were preheated and placed in a die at 680 °C ± 2 °C. Then, they were infiltrated by the squeeze-casting method with molten AlSn6Cu alloy. The process used a pressure of 80 MPa for 60 s. The molten alloy occupied the spaces in the salt preform, forming the AlSn6Cu-SiC skeleton. Cooling down was conducted at room temperature. Third, the removal of the salt space holder was conducted by using an ultrasonic cleaner (model UST28-200 B, Sofia, Bulgaria) filled with 79 °C distilled water. 

### 2.2. Characterization Methods

In this study, two types of specimens were tested: E and SE. E was the open-cell AlSn6Cu material (pore size 1000–1200 μm); SE was the open-cell AlSn6Cu-SiC composite (pore size 1000–1200 μm). A pin-on-disk system was used to perform dry-wear tests on all the test specimens with a Ducom Rotary tribometer, TR-20 Ducom model (Bangalore, India). The specimens had a spherical tip and were 20 mm high and 10 mm in diameter. They were formed by a lathe and underwent testing at a linear velocity of 1.0 m∙s^−1^ with two loads of 50 N and 100 N and a sliding distance of 420 m [27,34]. A disk of 140 mm diameter with a surface roughness of 1.6 Ra and a 62 HRC hardness, which was made from EN-31 steel, was the counter disk used for the wear experiments. The counterbody had the following concentrations, in wt.%: C 0.90–1.20; Si 0.10–0.35; Mn 0.30–0.75; Cr 1.00–1.60; Si 0.20; and Fe–rest. 

The cross-sections of the materials were tested for their average Vickers hardness (HV) using a light microscope (Polyvar Met, Reichert Jung, Wien, Austria). The light microscope was equipped with a semi-automatic micro-Vickers hardness tester (Micro-Duromat 5000 computer control, Reichert Jung, Wien, Austria). The test involved applying a force of 0.05 kg·f for 10 s and holding it for another 10 s.

The microstructure and phase composition of the open-cell AlSn6Cu-SiC composites were characterized by SEM, EDS, and XRD. The samples were prepared for characterization by polishing with different grades of emery paper and diamond paste, etching with Keller’s reagent, and coating with a thin layer of gold.

The SEM images were taken by a HIROX SH-5500 scanning electron microscope (SEM, Hirox Japan Co Ltd., Tokyo, Japan) with a QUANTAX 100 Advanced EDS system (EDS, BRUCKER Co., Frankfurt, Germany). The composite matrix and the SiC particles were analyzed by EDS to find out their elemental composition.

The phases in the composite matrix and the SiC particles were identified by XRD analysis. A powder X-ray diffractometer (BRUCKER Co., Karlsruhe, Germany) with a LynxEye solid-state position-sensitive detector and Ni-filtered Cu Kα radiation was used for this purpose. The XRD patterns were recorded in the 2θ range of 20° to 80° with a step size of 0.02° and a scan speed of 0.5°/min. The phase analysis was conducted using the 2021 release of the PDF-2 ICDD database and the DiffracPlusEVA software package (https://www.bruker.com/en/products-and-solutions/diffractometers-and-x-ray-microscopes/x-ray-diffractometers/diffrac-suite-software/diffrac-eva.html).

### 2.3. Machine Learning Models

The dataset used for this study consisted of open-cell AlSn6Cu-SiC composites subjected to pin-on-disk experiments. Six different ML models were trained and tested on this dataset to predict their COF based on their independent parameters (load, hardness of reinforcement, reinforcement, linear velocity, and hardness of matrix). Using a 60:20:20 ratio and a random seed of 42 for reproducibility, the dataset was divided into three sets: training, validation, and test. RF regressors were used as feature importance plots of predicting the COF and mass wear of the test materials under different loads. The performance metrics (R2 score, RMSE, MSE, and MAE) of the test and validation sets of each model were calculated. Each ML model was visualized by a scatter plot that showed the variation in the COF as a function of the sliding distance for both the actual and predicted values. The plot used four different colors and labels to distinguish between the test and validation sets and between the actual and predicted values. The models were:

- Extreme gradient boosting (XGBoost): A scalable and efficient implementation of gradient boosting trees that uses a regularized objective function to prevent overfitting and improve generalization [35]. The XGBoost library was used for this model [36]. The model was previously used for the COF prediction of open-cell AlSi10Mg-Al_2_O_3_ composite materials [23].
- Support vector regression (SVR): A type of support vector machine (SVM) that performs regression by finding a linear function that fits the data with a maximum margin while allowing some errors. The SVR class from the scikit-learn library was used for this model [37,38]. The model was previously used to predict the COF of an open-cell AlSi10Mg-SiC composite [24] and an Al-based composite reinforced with graphene [14] and for the prediction of the volume loss of AA7075/Al_2_O_3_ [12].
- Random forest (RF): An ensemble method that builds multiple decision trees and averages their predictions and outputs the average prediction of the individual trees. It introduces randomness in the tree construction and feature selection, which reduces the variance and improves the accuracy of decision trees [39]. This is because randomness helps to avoid overfitting and creates more diverse and uncorrelated trees, which can produce more robust and stable predictions. The RandomForestRegressor class from the scikit-learn library was used for this model. It was previously used for the prediction of the wear rate and COF of graphene-reinforced AMMCs [14] and for the prediction of the volume loss of ZK60/CeO_2_ composites [21].
- k-nearest neighbors (KNN): A non-parametric method that predicts the output of a new instance based on the k-nearest neighbors in the training set [40]. It is simple and effective for classification and regression problems, but it requires a distance metric to measure the similarity between instances. The KNeighborsRegressor class from the scikit-learn library was used for this model. The model was previously used to predict the COF and wear rate of an Al-based composite reinforced with graphene [14].
- Decision tree (DT): A simple and interpretable method that splits the data into homogeneous regions based on a series of rules [41]. It works with both numerical and categorical variables, but it tends to overfit and be unstable. The DecisionTreeRegressor class from the scikit-learn library was used for this model. DT was previously employed for the prediction of the volume loss of ZK60-CeO_2_ composites [21].
- Adaptive boosting (Adaboost): A boosting method that combines multiple weak learners (such as decision trees) into a strong learner by iteratively adjusting the weights of the training instances according to the errors of the previous learners [42]. It can enhance the precision and reliability of simple models, but it is affected by noise and outliers. The AdaBoostRegressor class from the scikit-learn library was used for this model. Adaboost was previously used for the prediction of the microhardness of different alloys and metal-based composite materials fabricated by laser powder bed fusion [43].

Section 2 described how the open-cell AlSn6Cu-SiC composites were fabricated in this study using liquid-state processing and subjected to dry-friction wear tests under different loads. The purpose of this study was to investigate the tribological behavior of open-cell composites with AlSn6Cu as a matrix material and compare it with previous studies of open-cell AlSi10Mg-Al_2_O_3_ and AlSi10Mg-SiC composites [26,27]. It was assumed that the fabricated AlSn6Cu-SiC composite would have a similar or better wear performance than the other composites. Section 3 presents the data obtained from the microstructural characterization and the wear tests and analyzes them using six ML models. The results show whether the assumption was valid and how the open-cell AlSn6Cu-SiC composite performed under the different loads.

## 3. Results and Discussion

### 3.1. Microstructure

**Figure 1:**
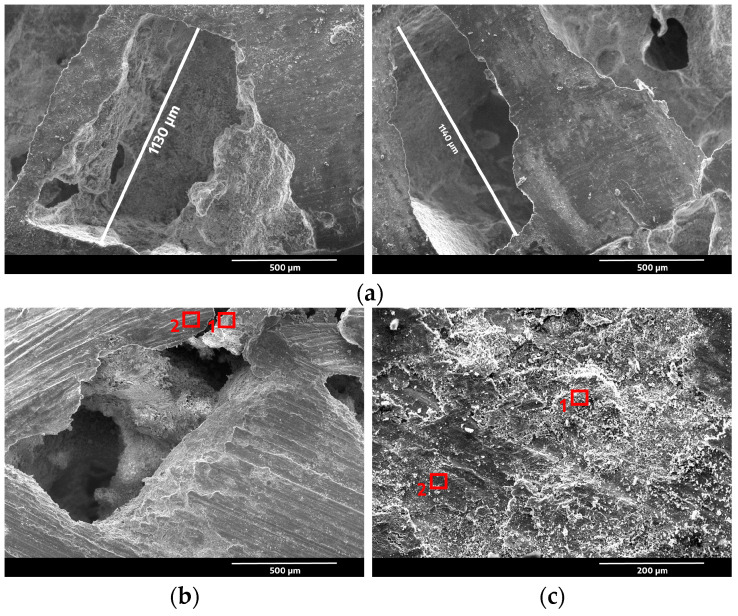
SEM images of open-cell AlSn6Cu-SiC composite: (**a**) before conducting tribological tests; (**b**) after conducting tribological tests at load of 50 N; and (**c**) after conducting tribological tests at load of 100 N.

**Figure 2:**
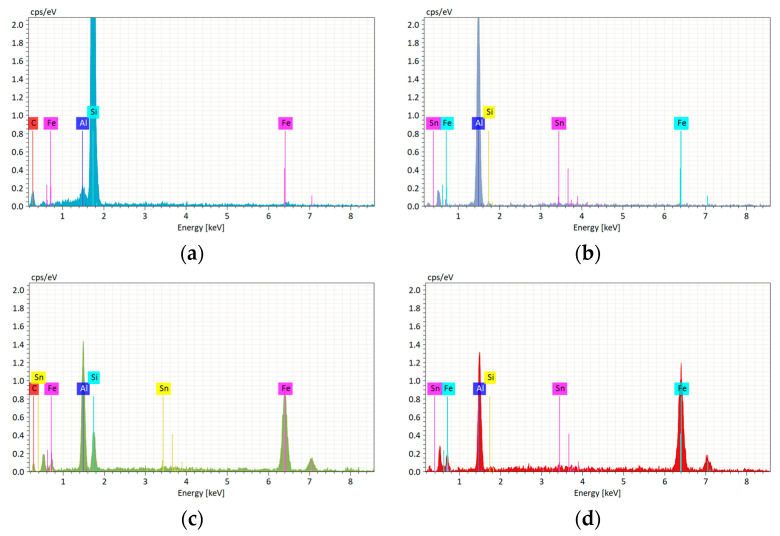
EDS spectra of open-cell AlSn6Cu-SiC composite after conducting tribological tests at different loads: (**a**) related to contact zone shown in Table 2, analysis 1, at load of 50 N; (**b**) related to contact zone shown in Table 2, analysis 2, at load of 50 N; (**c**) related to contact zone shown in Table 3, analysis 1, at load of 100 N; and (**d**) related to contact zone shown in Table 3, analysis 2, at load of 100 N.

To confirm the open-cell structure of the composites, we performed SEM analysis, as presented in Figure 1a. The SEM images showed that the composites had a porous structure and that the pores were mainly distributed between the size of the NaCl particles, i.e., 1000–1200 µm. Based on the SEM image of the contact surface shown in Figure 1b of the open-cell AlSn6Cu-SiC composite subjected to wear at a load of 50 N, the wear mechanism was mainly abrasive wear. It occurs when hard particles or asperities on sliding surfaces cause material removal by ploughing or cutting. Therefore, the steel counterbody acted as an abrasive agent and caused damage to the softer composite. Its surface roughness of 1.6 Ra could have also contributed to the abrasive wear by creating more contact points and friction between the sliding surfaces. The EDS analysis shown in Table 2 shows that zone 1 (Figure 2a) had a high concentration of Si and C, which indicates that it was a SiC particle. Zone 2 (Figure 2b) had a high concentration of Al and a low concentration of Sn, which indicates that it was part of the AlSn6Cu matrix. The SiC particles located in zone 1 presented in Figure 1a could reduce the mass loss of the composite by acting as a barrier to prevent the penetration and ploughing of the steel disk in addition to being a reinforcement to support the cell walls of the matrix and reduce its deformation. Based on the SEM image of the contact surface shown in Figure 1c of the open-cell AlSn6Cu-SiC composite subjected to wear at a load of 100 N, the wear mechanism was mainly abrasive wear. The EDS analysis shown in Table 3 shows that zone 1 (Figure 2c) had a moderate concentration of Si and C, which indicates that it was a SiC particle. Zone 2 (Figure 2d) had a low concentration of Si and C and high concentrations of Fe from the counterbody and Al from the matrix of the composite, which indicates that it was part of the AlSn6Cu matrix. The SEM images show that the composite had more scratches, grooves, and debris on its surface at higher loads, as shown in Figure 1c, which indicate that there was more material removal by abrasive wear. A pore could be seen on the surface of the AlSn6Cu-SiC composite after conducting the pin-on-disk test at a load of 50 N (Figure 1b) but not after conducting the test at a load of 100 N (Figure 1c). This was because the load affected the wear behavior of the composite, which influenced its surface morphology. As the load increased, the wear depth increased, which resulted in more material removal and more wear debris formation. The wear debris could accumulate on the surface and form a layer of compacted material, which could cover or fill the pores. The wear debris could also act as a third-body abrasive and cause more damage to the surface. The increased load also caused more contact pressure and friction between the sliding surfaces, which resulted in a higher temperature and wear rate. The higher temperature and wear rate could degrade the lubricating effect of the Sn phase and expose the harder SiC particles, which could increase the COF of the composite.

**Figure 3:**
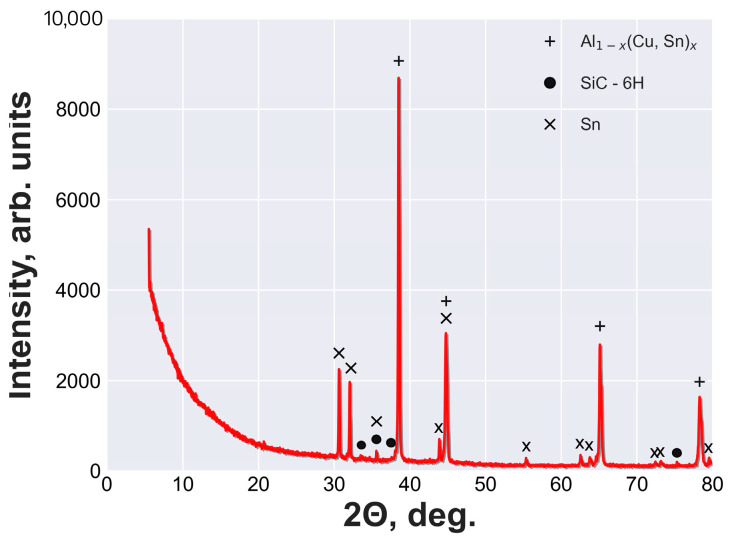
XRD patterns of open-cell AlSn6Cu-SiC composite. The peaks marked with + indicate the presence of Al_1−x_(Cu,Sn)_x_. The peaks marked with • indicate the presence of SiC-6H. The peaks marked with × indicate the presence of Sn.

The XRD patterns of the open-cell AlSn6Cu-SiC composite are shown in Figure 3. The phases in the composite matrix and the SiC particles were recorded in the 2θ range of 20° to 80° with a step size of 0.02° and a scan speed of 0.5°/min. The phase analysis was conducted using the 2021 release of the PDF-2 ICDD database. The main phases detected in the composite were Al, Sn, and SiC. Al_1−x_(Cu,Sn)_x_ was the main phase of the matrix and corresponded to the peaks at 2θ = 38.5°, 44.7°, 65.1°, and 78.2°. The Sn phase corresponded to the peaks at 2θ = 30.5°, 32.1°, 35.5°, 43.8°, 44.7°, 55.3°, 62.5°, 63.7°, 72.4°, 73.2°, and 79.4°. SiC–6H was the reinforcement phase and corresponded to the peaks at 2θ = 33.6°, 35.5°, 38.0°, and 75.3°.

### 3.2. Wear and Micro-Hardness Behavior

**Figure 4:**
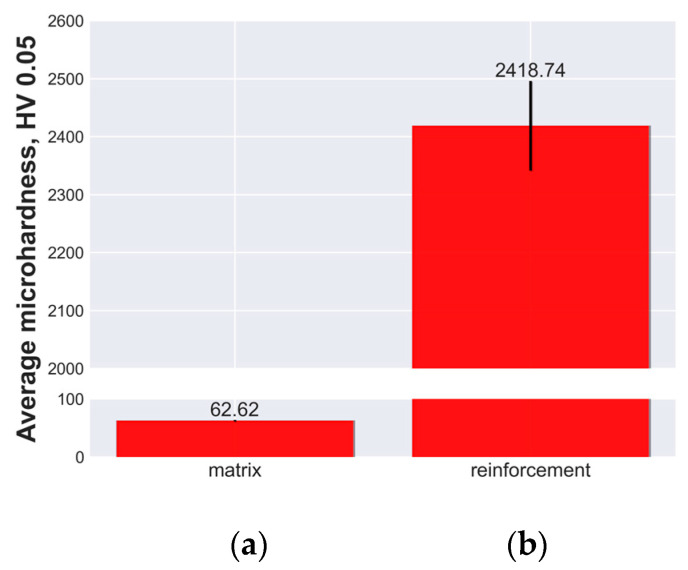
Micro-hardness tests of: (**a**) the matrix of open-cell AlSn6Cu; (**b**) the reinforcement of open-cell AlSn6Cu-SiC composite.

The cross-sections of the materials were tested for their average Vickers hardness (HV) using a force of 0.05 kg·f for 10 s and holding it for another 10 s, as shown in Figure 4. The results showed that the matrix of the open-cell AlSn6Cu had a microhardness value of 62.92 HV, while the reinforcement of the open-cell AlSn6Cu-SiC composite had a microhardness value of 2418.74 HV. 

**Figure 5:**
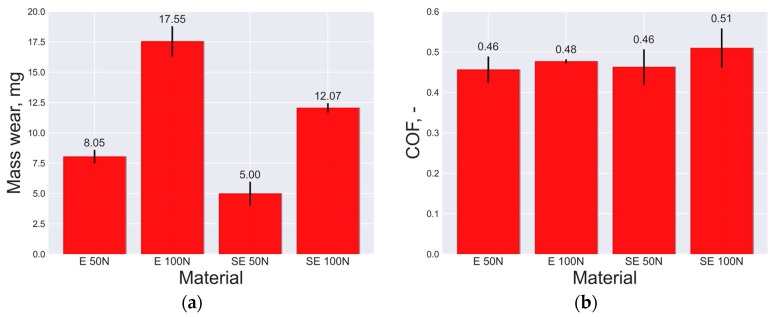
Wear behavior measured by pin-on-disk method of open-cell AlSn6Cu-SiC composite and open-cell AlSn6Cu material at loads of 50 N and 100 N: (**a**) mass wear results; (**b**) COF results.

The pin-on-disk method was used to perform the wear tests under dry-sliding conditions at room temperature with a sliding speed of 1.0 m∙s^−1^ and a sliding distance of 420 m. The materials tested were the open-cell AlSn6Cu material and the open-cell AlSn6Cu-SiC composite. The wear parameters measured were the mass loss and COF, which provided quantitative measures of the tribological behavior of the materials. Figure 5 displays the outcomes of the wear tests.

It was evident that the open-cell AlSn6Cu-SiC composite had an enhanced tribological behavior when compared with the open-cell AlSn6Cu material in terms of the mass wear, as presented in Figure 5a. At a load of 50 N, the mass wear of the composite decreased by 38% compared to the material. At a load of 100 N, the mass wear of the composite decreased by 31% compared to the material. The load increase had an effect on the mass wear of the composite, and a 7% percentage difference was observed (from 38% to 31%). The AlSn6Cu-SiC composite also had less variation in its mass loss values than the AlSn6Cu material, which means that it had a more stable and consistent behavior under the different load levels. The AlSn6Cu-SiC composite may be a more suitable material for applications that require a high resistance to friction and wear under varying load conditions. For the COF results, both materials had similar COF values, as can be seen in Figure 5b. At a load of 50 N, both materials had identical COF values. At a load of 100 N, the COF of the composite increased by 6% compared to the material, as can be seen in Figure 5b. The load increase had an effect on the COF of the composite, and a 6% percentage difference was observed. The COF of the composite increased with the increase in the load because the higher load caused more contact pressure and friction between the sliding surfaces, which resulted in a higher temperature and wear loss. The composite had an enhanced tribological behavior when compared with the material in terms of mass wear because the addition of the SiC particles could improve the wear resistance and reduce the mass loss of the composite. The SiC particles could act as a barrier to prevent the penetration and ploughing of the counterface in addition to acting as a reinforcement to support the cell walls of the matrix and reduce its deformation.

### 3.3. COF Prediction and Model Performance Evaluation

The ML models were evaluated using four performance metrics: R2 score, RMSE, MSE, and MAE. These metrics measured how well the ML models could predict the COF of the material based on the features [21]. The test and validation sets were different subsets of the data that were used to measure the performance of the ML models. The test set measured the final performance of the models, while the validation set adjusted the hyperparameters and chose the best model. The test set should reflect the real-world data distribution and should not be used for training or validation. 

**Figure 6:**
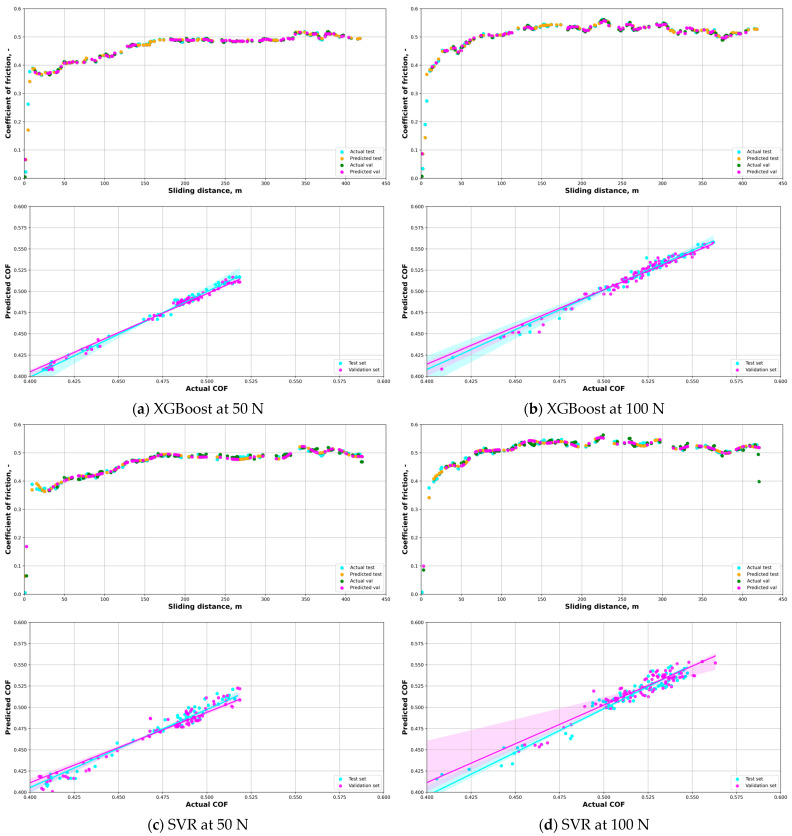

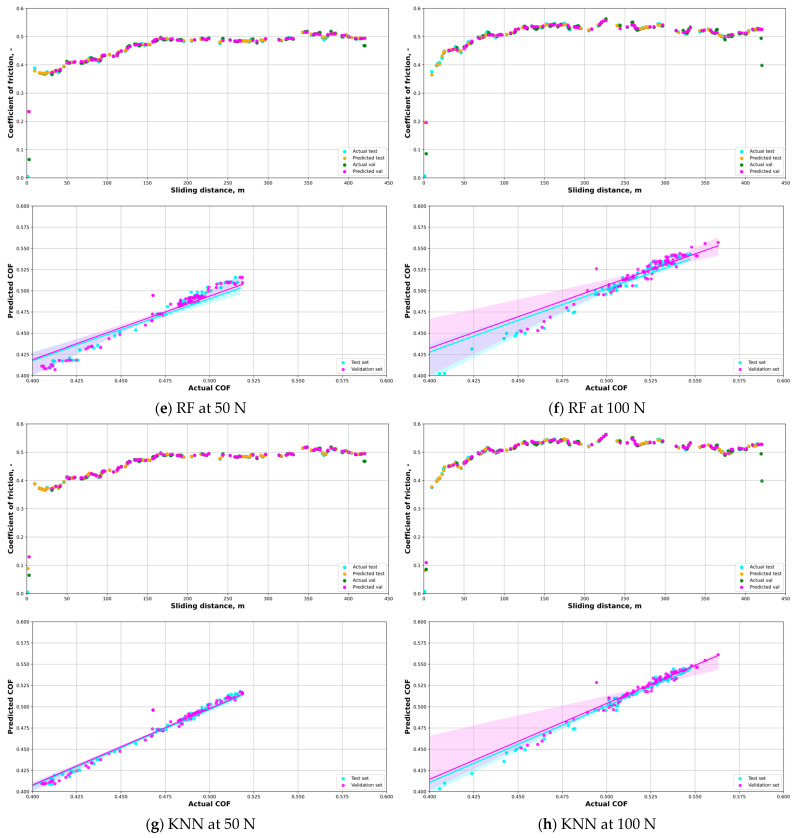

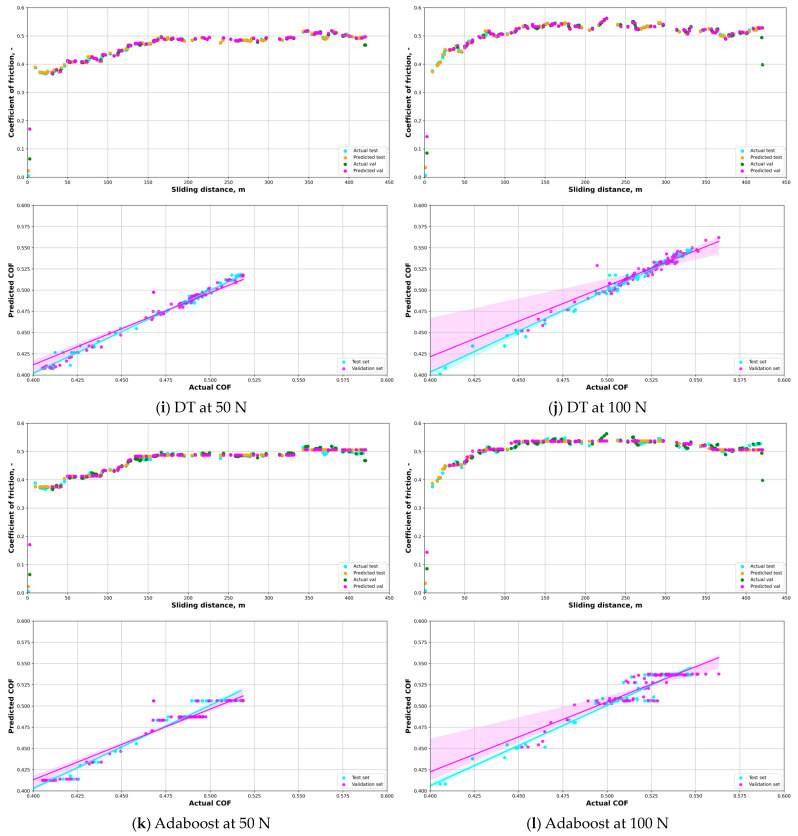
ML models used for prediction of the COF of open-cell AlSn6Cu-SiC composite at different loads: (**a**) XGBoost at 50 N; (**b**) XGBoost at 100 N; (**c**) SVR at 50 N; (**d**) SVR at 100 N; (**e**) RF at 50 N; (**f**) RF at 100 N; (**g**) KNN at 50 N; (**h**) KNN at 100 N; (**i**) DT at 50 N; (**j**) DT at 100 N; (**k**) Adaboost at 50 N; and (**l**) Adaboost at 100 N.

Based on the results of the test and validation sets shown in Table 4 and the plots shown in Figure 6, the best ML model for predicting the COF of the composite material under both loads was the DT model, as it had the highest R2 score (0.9965) and the lowest RMSE, MSE, and MAE scores for both sets. The DT model could explain more than 99% of the variance in the COF data and had very low errors in its predictions. The worst ML model for predicting the COF of the composite material under both loads was the RF model (0.8592), as it had the lowest R2 score and the highest RMSE and MSE scores for both sets. The RF model could only explain about 86% to 90% of the variance in the COF data and had relatively high errors in its predictions. The other ML models, such as XGBoost, SVR, KNN, and Adaboost, had intermediate performance metrics for predicting the COF of the composite material under both loads. They had R2 scores ranging from 0.9076 to 0.9883, RMSE scores ranging from 0.0072 to 0.0162, MSE scores ranging from 0.0001 to 0.0003, and MAE scores ranging from 0.0026 to 0.0090 for both sets.

The load variation had a substantial effect on the performance of some of the ML models. At a load of 50 N, the best ML model on both the test and validation sets was DT, with R2 scores of 0.9965 and 0.9518, respectively. DT had the highest accuracy and the lowest error among all the models at this load level. However, DT may also be prone to overfitting, which means that it may perform poorly on new or unseen data. At a load of 100 N, the best ML model on both the test and validation sets was XGBoost, with R2 scores of 0.9696 and 0.9769, respectively. XGBoost had a slightly lower accuracy but still a lower error than DT at this load level. XGBoost is an effective ensemble method that uses many decision trees to enhance its performance and lower its variance. XGBoost may be more robust and stable than DT in handling complex data and noise.

The ML model that had the least load variation effect on its performance was KNN, with R2 score differences of 0.0029 and 0.0619, RMSE differences of 0.0008 and 0.0065, MSE differences of 0 and 0.0001, and MAE differences of 0.0005 and 0.0015 between the two load levels on both the test and validation sets, respectively. This means that KNN had a consistent accuracy and error across the different load levels, which may indicate that it was not sensitive to the load variation in the data. The ML model that had the most load variation effect on its performance was DT, with R2 score differences of −0.0426 and −0.0453, RMSE differences of −0.0012 and −0.0038, MSE differences of 0 and −0.0001, and MAE differences of −0.0007 and −0.0016 between the two load levels on both the test and validation sets, respectively. This means that DT had a lower accuracy and higher error at a higher load level than at a lower load level, which may indicate that it was overfitting the data at the lower load level and not generalizing well at the higher load level.

These ML models could estimate the COF of the composites accurately and efficiently without expensive and time-consuming wear tests. They could also predict the COF of the composites under the different conditions or scenarios without physical models or assumptions, which could reduce the error and increase the scope of the tribological analysis.

### 3.4. Feature Importance Analysis for Mass Wear and COF Prediction

**Figure 7:**
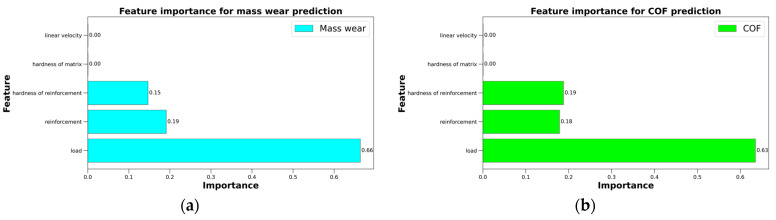
Feature importance plots for predicting (**a**) mass wear and (**b**) COF.

To investigate the importance of different independent variables for predicting the mass wear and COF of the open-cell AlSn6Cu-SiC composites and the open-cell AlSn6Cu materials under different loads, RF regressors were used as a feature selection method. The feature importance attribute of RF regressors assigns values that are proportional to the average reduction in variance that each feature brings to the decision trees in the ensemble. The values are then normalized by dividing by the sum of all the values so that they add up to 1. A high value indicates a high importance of a feature in the output prediction, but it does not necessarily mean a high correlation or causation between the feature and the output. The entire dataset of all the specimens was used, and their parameters (load, hardness of reinforcement, reinforcement, linear velocity, and hardness of matrix) were used to fit two RF regressors: one for mass wear and one for COF. The feature importance values were then obtained from both regressors and sorted in descending order for both targets. The results are shown in Figure 7. The most important feature for both the mass wear and COF prediction was the load, as it had the highest importance value of 63% for the COF plot and 66% for the mass wear plot. This means that the load had a strong influence on the wear behavior of the materials. The hardness of reinforcement was the second-best feature for the COF prediction, as it had a high importance value of 19% for both loads. This means that the hardness of reinforcement had a significant impact on the friction behavior of the material, as it affected the resistance to abrasion, ploughing, and grooving by the harder particles. The reinforcement was the second-best feature for the mass wear prediction, as it had a high importance value of 19% for both loads. This means that the reinforcement had a significant impact on the wear behavior of the tested material, as it affected the removal and transfer of material from the sliding surfaces. The presence of reinforcement reduced the mass wear, as the particles reinforced the cell walls, protected the matrix from severe wear, and acted as a self-lubricating layer.

In this study, open-cell AlSn6Cu-SiC composites were fabricated using liquid-state processing, and they were subjected to dry-friction wear tests. To the best of our knowledge, this is the first study that has investigated the tribological behavior of open-cell composites (pore size 1000–1200 μm) with AlSn6Cu as a matrix material. Based on previous studies of open-cell AlSi10Mg-SiC [27] and AlSi10Mg-Al_2_O_3_ [26] composites produced by squeeze-casting and subjected to dry-friction wear tests, which have reported significant improvements in mass wear (53.5–65.7%) and insignificant improvements in COF (3.2–4.8%), it was assumed that the reported composites were in the same range of wear improvement. This study demonstrated that the open-cell AlSn6Cu-SiC composite had a superior wear behavior and a reduced mass loss compared to the open-cell AlSn6Cu material under different loads while maintaining similar COF values. The results also suggested that the ML models used in this study could provide accurate and reliable predictions of the COF of the materials based on their features, but they could also be sensitive to data quality and quantity, overfitting or underfitting, and load variation.

## 4. Conclusions

This paper investigated the tribological behavior of open-cell AlSn6Cu-SiC composites under different loads (50 N and 100 N) using the pin-on-disk method. The composites were fabricated by squeeze-casting. This study is the first to investigate the tribological behavior of open-cell AlSn6Cu-SiC composites under different loads using the pin-on-disk method and ML models. This study provides new insights into the effects of load, hardness of reinforcement, reinforcement, linear velocity, and hardness of matrix on the mass wear and COF of these composites. Thid study also demonstrates the potential of ML models to accurately and reliably predict the COF of these composites based on their features. The results showed that the open-cell AlSn6Cu-SiC composite had an enhanced tribological behavior compared to the open-cell AlSn6Cu material in terms of mass wear while maintaining the COF at the same level. The mass wear of the composite decreased by 38% at a load of 50 N and by 31% at a load of 100 N compared to the unreinforced material. The COF of the composite increased by 6% at a load of 100 N compared to the unreinforced material, while it was identical to the material at a load of 50 N.

ML models, such as DT, RF, XGBoost, SVR, KNN, and Adaboost, were used to predict the COF vs. the sliding distance of the composite materials. The best ML model for both load levels was DT, which had the highest R2 score and the lowest RMSE, MSE, and MAE scores. The worst ML model for both load levels was RF, which had the lowest R2 score and the highest RMSE and MSE scores. The other ML models, such as XGBoost, SVR, KNN, and Adaboost, had intermediate performance metrics. The load variation had a significant effect on some ML models, such as DT, which had a lower accuracy and a higher error at the higher load level than at the lower load level. The ML model that had the least load variation effect was KNN, which had a consistent accuracy and error across the different load levels. These results suggest that some ML models are more robust and stable than others in handling complex data and noise at different load levels.

To investigate the importance of different independent variables for predicting the mass wear and COF of open-cell AlSn6Cu-SiC composites and open-cell AlSn6Cu materials under different loads, RF regressors were used as a feature selection method. Based on the results of the method, the most important feature for both the mass wear and COF predictions was the load, as it had the highest importance value for both plots. The hardness of reinforcement was the second-best feature for the COF prediction, while reinforcement was the second-best feature for the mass wear prediction. This study has some limitations that should be addressed in future research. This study only used two load levels (50 N and 100 N) and one sliding velocity (1.0 m∙s^−1^) for the tribological tests. Future studies could use more load levels and sliding velocities to explore the tribological behavior of the composites under different conditions. The current study only used one type of reinforcement (SiC) and one type of matrix (AlSn6Cu) for the composites. Future studies could use different types of reinforcements and matrices to investigate their effects on the tribological behavior of the composites. Future studies could employ deep learning methods to increase the data size and dimensionality and enhance the performance and robustness of the COF prediction.

## Figures and Tables

**Table 1:** AlSn6Cu alloy composition.

Element	Sn	Cu	Ni	Si	Fe	Mn	Zn	Mg	Ti	Al
Concentration, wt.%	5.5–6.5	1.3–1.7	0.2	0.3	0.4	0.2	0.2	0.1	0.05–0.2	remainder

**Table 2:** EDS analysis of the contact surface of open-cell AlSn6Cu-SiC composite after conducting tribological tests at load of 50 N in selected zones from Figure 1b.

Analysis No.	Si	C	Fe	Al	Sn
1	61.91	35.13	1.50	1.46	-
2	0.77	-	0.58	97.88	0.76

**Table 3:** EDS analysis of the contact surface of open-cell AlSn6Cu-SiC composite after conducting tribological tests at load of 100 N in selected zones from Figure 1c.

Analysis No.	Si	C	Fe	Al	Sn
1	9.85	9.30	47.91	30.62	2.32
2	0.42	-	63.914	33.33	2.34

**Table 4:** Performance metrics of all ML methods.

ML Method	Load (N)	Set	R2 Score	RMSE	MSE	MAE
XGBoost	50	Test	0.9678	0.0119	0.0001	0.0040
XGBoost	50	Val	0.9877	0.0072	0.0001	0.0030
XGBoost	100	Test	0.9696	0.0132	0.0002	0.0051
XGBoost	100	Val	0.9769	0.0095	0.0001	0.0042
SVR	50	Test	0.9803	0.0093	0.0001	0.0063
SVR	50	Val	0.9468	0.0131	0.0002	0.0067
SVR	100	Test	0.9814	0.0088	0.0001	0.0071
SVR	100	Val	0.9195	0.0151	0.0002	0.0077
RF	50	Test	0.8592	0.0250	0.0006	0.0052
RF	50	Val	0.8880	0.0190	0.0004	0.0050
RF	100	Test	0.8969	0.0208	0.0004	0.0054
RF	100	Val	0.8712	0.0191	0.0004	0.0065
KNN	50	Test	0.9804	0.0093	0.0001	0.0026
KNN	50	Val	0.9775	0.0085	0.0001	0.0031
KNN	100	Test	0.9819	0.0087	0.0001	0.0033
KNN	100	Val	0.9200	0.0150	0.0002	0.0046
DT	50	Test	0.9965	0.0039	0.0000	0.0026
DT	50	Val	0.9518	0.0125	0.0002	0.0037
DT	100	Test	0.9939	0.0051	0.0000	0.0033
DT	100	Val	0.9065	0.0163	0.0003	0.0053
Adaboost	50	Test	0.9883	0.0072	0.0001	0.0056
Adaboost	50	Val	0.9376	0.0142	0.0002	0.0070
Adaboost	100	Test	0.9798	0.0092	0.0001	0.0068
Adaboost	100	Val	0.9076	0.0162	0.0003	0.0090

## Data Availability

Not applicable.
